# Change in health status in COPD: a seven-year follow-up cohort study

**DOI:** 10.1038/npjpcrm.2016.73

**Published:** 2016-10-20

**Authors:** Josefin Sundh, Scott Montgomery, Mikael Hasselgren, Mary Kämpe, Christer Janson, Björn Ställberg, Karin Lisspers

**Affiliations:** 1 Department of Respiratory Medicine, School of Medical Sciences, Örebro University, Örebro, Sweden; 2 Clinical Epidemiology and Biostatistics, School of Medical Sciences, Örebro University, Örebro, Sweden; 3 Clinical Epidemiology Unit, Department of Medicine, Karolinska Institutet, Stockholm, Sweden; 4 Department of Epidemiology and Public Health, University College, London, UK; 5 School of Medical Sciences, Örebro University, Örebro, Sweden; 6 Department of Medical Sciences, Respiratory, Allergy and Sleep Research, Uppsala University, Uppsala, Sweden; 7 Department of Public Health and Caring Science, Family Medicine and Preventive Medicine, Uppsala University, Uppsala, Sweden

## Abstract

Health status is a prognostic factor included in the assessment of chronic obstructive pulmonary disease (COPD). The aim of our study was to examine the associations of clinical factors with change in health status over a 7-year follow-up period. In 2005, 970 randomly selected primary and secondary care patients with a COPD diagnosis completed questionnaires including the Clinical COPD Questionnaire (CCQ); and in 2012, 413 completed the CCQ questionnaire again. Linear regression used difference in mean total CCQ score between 2005 and 2012 as the dependent variable. Independent variables were CCQ score at baseline 2005, sex, age, educational level, body mass index (BMI), smoking status, heart disease, diabetes, depression, number of exacerbations in the previous 6 months, dyspnoea (modified Medical Research Council (mMRC)). Health status worsened from mean total CCQ (s.d.) 2.03 (1.26) in 2005 to 2.16 (1.37) in 2012 (*P*=0.011). In linear regression with adjustment for baseline CCQ; older age, lower education, higher mMRC and BMI below 25 kg/m^2^ at baseline were associated with worsened health status in 2012. When sex, age and all statistically significant measures were included simultaneously in the analysis of the main study group, higher mMRC and BMI below 25 kg/m^2^ were were associated with deteriorated health status (*P*<0.0001). A higher level of dyspnoea and lower weight were associated with worse health status in COPD. Strategies for decreasing dyspnoea and awareness of the possible increased risk of worsening disease in under- and normal-weight COPD patients are clinically important.

## Introduction

According to the updated GOLD (Global Initiative for Chronic Obstructive Lung Disease) recommendations, the assessment of disease severity in chronic obstructive pulmonary disease (COPD) patients should include lung function, exacerbation frequency and health status.^1^ Low health status is known to be associated with increased risk for re-admission to hospital and increased mortality.^2–4^ The importance of health status in COPD was emphasised in a recent study, where patients with a 1-year improvement or stable health status as assessed by the St Georges Respiratory Questionnaire (SGRQ) had a lower risk of exacerbations, COPD-related hospital admission or death.^5^

GOLD recommends the use of concise health status instruments for clinical practice,^6^ such as the COPD Assessment Test (CAT)^7^ or the Clinical COPD Questionnaire (CCQ).^8^ CCQ was introduced in 2003, 6 years before CAT, which makes it possible to examine the changes in health status over a longer time period. It has been shown to detect improvement in health status following pulmonary rehabilitation after acute exacerbations.^9,10^

To prevent deterioration, or even better facilitate improvement in health status, it is important to identify the factors influencing change in health status over time. The aim of our study was to examine the associations of different clinical factors with subsequent change in health status measured by CCQ over a 7-year follow-up period, in a Swedish multi-centre study including primary and secondary care patients with COPD.

## Results

Patient characteristics at baseline are shown in Table 1. In secondary care, statistically significantly more patients were never or ex-smokers, had ⩾2 exacerbations and COPD stage III or IV. The mean CCQ and modified Medical Research Council (mMRC) scores were higher indicating worse health status and dyspnoea in secondary care patients. Between 2005 and 2012, there was statistically significant worsening of the average health status in the study population (Table 2). For 42.9% of the patients, health status improved, for 51.1% of the patients health status worsened, and for 6.0% the score was unchanged after 7 years. Using the minimal clinical important difference of 0.4; 30% improved, 36.8% worsened and 32.2% had unchanged health status. For the domains, the mean functional status worsened, while no statistically significant changes were found for the domains of symptoms and mental state (Table 2).

**Figure 1:**
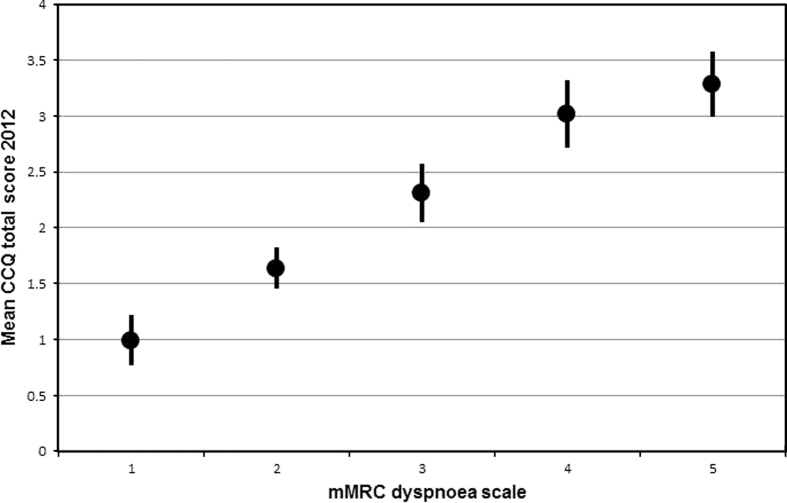
Dyspnoea and health status. Mean CCQ total scores in 2012 distributed over mMRC dyspnoea scale grades. CCQ, Clinical COPD Questionnaire; mMRC, modified Medical Research Council.

**Figure 2:**
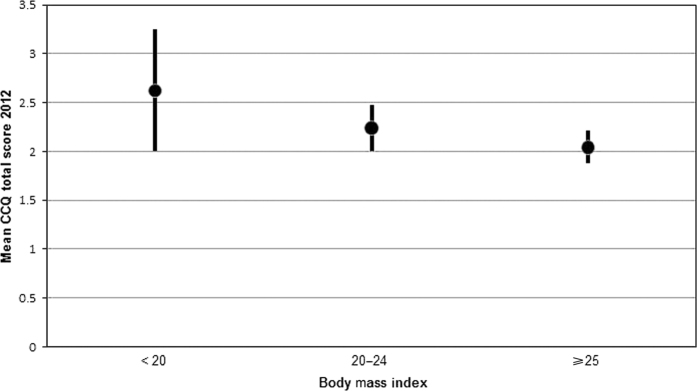
Body mass index and health status. Mean CCQ total scores in 2012 distributed over BMI groups. BMI, body mass index; CCQ, Clinical COPD Questionnaire.

Linear regression with the independent measures entered individually but always adjusted for baseline CCQ showed statistically significant associations with worsened health status at the follow-up for higher mMRC, underweight and normal weight compared with BMI ≥25 kg/m^2^, and higher COPD stage at baseline, and with improved health status at the follow-up for younger age, and higher education at baseline. The final multivariable analyses of the main study patient group showed that higher mMRC and underweight and normal weight compared with BMI ≥25 kg/m^2^ at baseline were statistically significantly associated with worsened health status at the follow-up (Table 3, Figures 1 and 2). The results of the regression analyses were substantially the same when repeated with difference in CCQ mean score between 2005 and 2012, or with CCQ mean score at follow-up 2012 as dependent variables. Multiple logistic regression analysis showed that higher mMRC score (odds ratio (95% confidence interval) 1.53 (1.21 to 1.94), *P*<0.0001), underweight (1.79 (1.11 to 2.87), *P*=0.016) and normal weight (2.54 (1.10 to 5.88), *P*=0.029) were statistically significantly associated with clinically relevant deterioration of health status.

In the multiple linear regression analyses with domain scores as dependent variables and adjustment for the same variables as in the main analysis; higher mMRC was associated worsened health status in all separate domains at the follow-up. Normal and underweight compared with BMI ≥25 kg/m^2^ were associated with worsened functional state, and normal weight with worsened symptom domain at the follow-up. In addition, female sex was associated with worse mental state, and younger age was associated with improved functional state at the follow-up (data not shown).

The analyses stratified by sex showed that the CCQ mean total score, the symptoms score and the functional state score increased in women, indicating deterioration of health status (Table 2). In the stratified multivariable regression analyses, dyspnoea was associated with worse health status at the follow-up in total and all domains in both men and women (data not shown). Among women, a statistically significant association was shown for normal weight compared with BMI ≥25 kg/m^2^ at baseline with worse functional state at the follow-up (data not shown). The interaction analyses showed no statistically significant effect modification by sex for any of the associations.

## Discussion

### Main findings

The first important finding of this multi-centre 7-year follow-up study of both primary and secondary care patients with COPD is that health status in COPD worsens over time. The mean change is below the minimal clinically important difference, but over a third of the study population had a clinically significant deterioration.

The second important finding of this study is that dyspnoea assessed by the mMRC scale and normal and underweight at baseline were associated with worsened health status at follow-up. The results of the linear regression analyses were substantially the same irrespective of whether difference in CCQ between 2005 and 2012 or CCQ at follow-up was used as the dependent variable. The results were also confirmed in multiple logistic regression of clinically significant deterioration in health status.

### Interpretation of findings in relation to previously published work

The finding that health status in COPD worsens over time is consistent with several studies where the mean SGRQ score worsened over a period of 4 years in patients with COPD.^11–13^ However, none of these studies included primary care patients or analyses of how factors other than smoking and lung function were associated with the deterioration. Another study, of only primary care patients with COPD, did not find that health status assessed by Euroqol-Five-Dimensions changed during a year.^14^ We speculate that the reasons that our study showed a clear worsening of health status could be that our study included both primary and secondary care patients, had a longer follow-up period and used a disease-specific instrument. Another study, using SGRQ for the assessment of health status in COPD patients in primary care, established that during the 1-year follow-up, health status improved in a third of the patients, worsened in a third and was unchanged in a third.^15^ In our study, there was a corresponding distribution of improvement and worsening of health status, although fewer patients had unchanged score probably reflecting a longer follow-up time and possibly also the different properties of the SGRQ and CCQ. We find it clinically important that the changes in health status were demonstrated by a brief to administer and easy to use instrument.

Dyspnoea has previously been presented to predict change in SGRQ in COPD in a 1-year follow-up period, although the results were not repeated in the corresponding conditional change model.^5^ Our finding that dyspnoea at baseline was associated with worsened health status remained even in the conditional change model and with further adjustment for confounders. Interestingly, the predicting property of dyspnoea for change in health status in COPD has been examined for two different multidimensional indices, both including dyspnoea assessed by the mMRC. The DOSE (dyspnoea, obstruction, smoking, exacerbation) index^16^ was found to predict changes in CCQ in a 2-year follow-up period,^17^ and the BODE (BMI, obstruction, dyspnoea, exercise capacity) index^18^ predicted change in SGRQ in a 3-year follow-up period.^19^ In the latter study, the composite BODE index was also replaced by the included mono-components, with the result that dyspnoea and exacerbations but not BMI predicted change in health status.^19^

It is not surprising that dyspnoea was associated with the symptom score, as dyspnoea in itself is one of the items in this domain. However, it is most interesting that dyspnoea were associated with all the three domains of CCQ, and actually with higher amplitudes for the other two domains. This shows that dyspnoea is indeed of importance for all aspects of health status. The mental state domain score differed from the other domains and from the total score, by being the only domain with an unchanged health status score during the follow-up period. In male patients, there was a tendency towards improvement, in accordance with the significant association of male sex with improved mental health status in the multivariate regression analysis.

Our finding of an association between BMI and health status is consistent with a recent study of smokers where weight gain was associated with worsening of health status as measured by SGRQ in obese patients but with improvement of SGRQ score in normal-weight smoker, over a median follow-up time of six years.^20^ However, in that study of only a fourth of the patients had COPD, so our results extend the knowledge to include a clinical COPD population with all the stages of COPD. We are not aware of any previous longitudinal studies where normal and underweight or mMRC were associated with health status as assessed by the CCQ or any other such concise instrument, and no studies with a follow-up time as long as 7 years. Again, we find it important that our study shows that these associations are picked up even by a shorter and clinically more useful instrument.

### Strengths and limitations of this study

The strengths of our study are that it is longitudinal over a period of 7 years, that it uses a multi-centre design and that both primary and secondary care patients are included. A limitation is that spirometry data were not available for the whole study population. This could be due to the fact that spirometry is not always regularly repeated in clinical management of COPD.^21^ However, the aim of our study was to perform a real-life study of a clinical COPD population, which ensures a high generalisability of the results. Indeed, the results of the subgroup multivariable regression analysis were substantially the same as in the main analysis, although not statistically significant probably owing to reduced power. In the TORCH study, Jones *et al*.^22^ found that COPD stage predicted a faster decrease of SGRQ score. The fact that we did not find an association with lung function in our study, could also be due to reduced power with available spirometry data in only a subgroup of patients or select effects. Another potential limitation is that spirometry data were collected during the period of 2000 to 2003, before the completion of patient questionnaires in 2005. We believe that lung function did not change enough in this short period to influence the results spuriously. Associations of exacerbation frequency and lung function with impaired health status have been shown in previous studies.^23,24^ The fact that exacerbation frequency was not associated with HRQL in this longitudinal study could be due to our definition, which also included milder exacerbations, or to the fact that the number of exacerbations over the past 6 months was used. The reason for this procedure was that the information was obtained from patient questionnaires, where a longer period would increase the risk of recall bias. We found no associations for current smoking with health status. We speculate that this is due to the reference group of not current smoking including both never and ex-smokers, where the latter are actually those with most impaired health status and who have stopped smoking. In addition, even if tobacco smoking is the major cause of COPD, it is rather the subsequent obstruction than the smoking in itself that causes low health status.

Weight and height were self-reported as too few records contained those data, possibly causing bias. However, we analysed the self-reported and objectively measured values in the population with both values available (*n*=132), and found no statistically significant differences. Measuring change between two time points can be problematic as the measure at baseline can influence the likelihood of change, for example, a unit change from a low or high starting point may have different consequences. Therefore, we used conditional change modelling so that both the relative and absolute scores of the health status variables are taken into account.

The GOLD strategy document^1^ recommends the use of CAT^7^ and alternatively the CCQ^8^ as suitable short health status instruments in COPD. Both instruments correlate well with the GOLD standard health status instrument in COPD; SGRQ.^7,8^ The reason for using CCQ in our study was the longer period of access for clinical use, as CAT was not accessible in 2005. Future longitudinal studies of CAT would be of interest. However, in our opinion, CCQ is a very good substitute, as a previous cross-sectional study has shown that similar clinical factors influence the two instruments.^25^

### Implications for future research, policy and practice

Important clinical applications of our study could be to find strategies for the treatment of dyspnoea and for gaining weight in under- and normal-weight patients with COPD. Fortunately, a Cochrane review has showed that nutritional support in malnourished COPD lead to weight gain and improved health status as measured by SGRQ.^26^ Moreover, several Cochrane meta-analyses have established that both pharmacological treatment with bronchodilators^27,28^ and pulmonary rehabilitation^29^ can reduce dyspnoea in patients with COPD. Thus, examining BMI and grade of dyspnoea in the management of both primary and secondary care COPD patients is of great importance for identifying factors possible to modify in order to improve health status.

## Conclusions

We conclude that health status worsens over time, and that higher level of dyspnoea and lower BMI at baseline are associated with worsened health status at a 7-year follow-up in COPD. Strategies for decreasing dyspnoea and awareness of the possible increased risk of worsening disease among underweight and normal-weight patients with COPD might be of clinical importance.

## Materials and Methods

### Data collection

**Figure 3:**
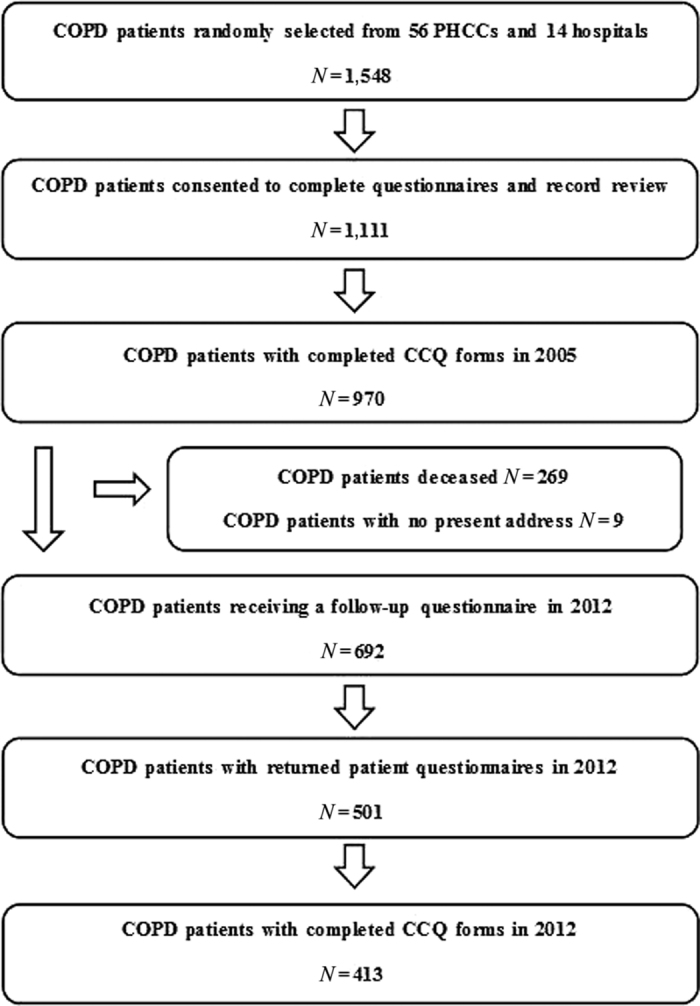
Flow chart. CCQ, Clinical COPD Questionnaire; COPD, chronic obstructive pulmonary disease; PHCC, primary health care centre.

In 2005, the PRAXIS study cohort was created, with primary and secondary care COPD patients from seven county councils in central Sweden.^4,25,30–32^ Each county council was represented by the department of respiratory medicine in their central hospital, the department of internal medicine from one randomly selected district hospital and eight randomly selected primary health care centres (PHCCs), in total 14 hospitals and 56 PHCCs. A list of all the patients aged 18–74 years with a doctors diagnosis of COPD (ICD-10 code J44) in the medical records during the period of 2000–2003 was compiled for every participating centre. A centralised random selection recruited 1,548 patients, including 1,084 in primary care and 464 in hospital clinics. The data were collected through patient questionnaires and record reviews for a total of 1,111 patients. The patient questionnaires included questions about patient characteristics and the Swedish version of CCQ. The number of patients with fully completed CCQ items in 2005 was 970. A paper presenting the associations with CCQ mean values in this study group was published in 2011.^33^ In 2012, a similar follow-up questionnaire was sent to the remaining 692 patients. The main study group included the 413 patients that returned a questionnaire with fully completed CCQ items (Figure 3).

### Patient characteristics and measures

Information on age, sex, smoking habits, level of education, number of exacerbations, height, weight, health status assessed by CCQ and grade of dyspnoea as measured by the mMRC scale^34^ was provided by the patient questionnaires. The exacerbations were defined as emergency visits during the last 6 months owing to deterioration in lung disease, with the answer choices 0, 1, 2 or >2 in the questionnaire. Information on spirometry data and presence of heart disease, diabetes and depression was obtained from the review of records for the period of 2000 to 2003. Heart disease and diabetes mellitus were defined as having the diagnoses of ischaemic heart disease or heart failure and diabetes type 1 or type 2 recorded anytime during the period of 2000 to 2003. Depression was defined as having a diagnosis of depression in combination with antidepressant drug treatment during the period of 2000 to 2003. Age from the 2005 questionnaire was categorised as ⩽60, 61–70 and >70 years. The dichotomous educational variable identified the most highly educated group as those who had continued in full-time education for at least 2 years beyond the Swedish compulsory school period of 9 years. Smoking history was categorised into current smoking, ex-smoking, occasional smoking and never smoking. Obesity was defined as body mass index (BMI) ⩾30, overweight as BMI <30 and ⩾25, and underweight as BMI <20 kg/m^2^, and BMI was categorised as overweight or obesity (BMI ≥25), normal weight (BMI 20–24.9), and underweight as BMI <20 kg/m^2^. The number of exacerbations over the previous 6 months were grouped as 0, 1 or ⩾2. In patients where spirometry data were available (*n*=237), their disease was graded on the basis of forced expiratory volume in 1 s (FEV_1_; ref. 1) expressed as a percentage of the European Community for Steel and Coal reference values (FEV_1_% predicted).^35^

### The CCQ

The CCQ consists of 10 questions distributed in three domains: symptoms, mental state and functional state. The observed symptoms are dyspnoea, cough and phlegm; mental state includes questions about feeling depressed and concerns about breathing; and functional state describes limitations in different activities of daily life owing to lung disease. The questions are assessed by a seven-point scale from zero to six.^8^ There are two versions of the CCQ, one where the questions apply to the previous week and one to the previous 24 h. This study used the version referring to the previous week. The main outcome measure is the mean CCQ value, with separate scores for each domain and a higher value indicates lower health status.^8^ The minimal clinical important difference for CCQ is established to be 0.4 units.^36,37^ In the present study, total mean CCQ scores and CCQ mean domain scores for 2005 and 2012 were calculated.

### Statistical analysis

The analyses were performed using SPSS version 22.0 (SPSS Inc, Chicago, IL, USA). The CCQ total mean difference between 2005 and 2012 was calculated. The Chi-squared test or Student's *t*-test were used to examine the differences in patient’s characteristics between primary and secondary care patients, and Student’s *t*-test to examine differences in CCQ total score and domain scores between 2005 and 2012. The linear regression analysis was used to examine differences in CCQ total mean and domain mean scores, as well as CCQ total mean and domain mean scores between 2005 and 2012. All the analyses were performed unadjusted, adjusted for only CCQ score at baseline in 2005, and in addition with inclusion of the other measures singly or simultaneously. Age, sex, level of education, smoking status (four groups), exacerbation frequency over the last 6 months (three groups), heart disease, diabetes and BMI (three groups) were used as independent variables. Age, smoking status and BMI groups were modelled as series of binary dummy variables. In multivariable analysis; sex and the variables with statistically significant associations (age, educational level, BMI and mMRC) when entered singly were included. The effect of COPD stage was studied in the subgroup with available lung function data (*n*=237). Stratification and interaction analyses were used to investigate potential effect modification by sex. The interaction analyses adjusted for the main effects, using interaction terms for sex with each relevant variable and with further adjustment for the same factors as in the main multivariable regression analysis. Finally, the main analysis was performed as a logistic regression with deterioration in CCQ equal or greater than the minimal clinical important difference of 0.4 from 2005 to 2012 as the dependent variable, with adjustment for baseline CCQ score in 2005, sex, age, educational level, BMI and mMRC dyspnoea scale.

### Ethics

The study was approved by the Regional Ethical Review Board of Uppsala (Dnr 2010/090). Written consent was obtained for all the participating patients.

## Figures and Tables

**Table 1:** Patient characteristics at baseline

*Patient characteristics*	*Primary care*	*Secondary care*	P*-value*
*Sex*			
Male	125 (40.6%)	46 (43.8%)	Ref
Female	183 (59.4%)	59 (56.2%)	0.562
			
*Age*			
⩽60	102 (33.1%)	34 (32.4%)	0.724
61–70	155 (50.3%)	56 (53.3%)	0.536
>70	51 (16.6%)	15 (14.3%)	Ref
			
*Educational level*			
Low	193 (63.3%)	65 (61.9%)	Ref
High	112 (36.7%)	40 (38.1%)	0.802
			
*Smoking habits*			
Never	21 (6.8%)	11 (10.5%)	0.023
Ex	183 (59.4%)	72 (68.6%)	0.013
Occasional	17 (5.5%)	6 (5.7%)	0.234
Current	87 (28.2%)	16 (15.2%)	Ref
			
*BMI*			
Underweight	21 (7.0%)	9 (8.7%)	0.559
Normal weight	97 (32.2%)	32 (31.1%)	Ref
Overweight	124 (41.2%)	40 (38.8%)	0.935
Obesity	59 (19.6%)	22 (21.4%)	0.704
			
*Number of exacerbations recent 6 months*			
0	226 (73.4%)	63 (60.0%)	Ref
1	45 (14.6%)	13 (12.4%)	0.918
⩾2	37 (12.0%)	29 (27.6%)	<0.0001
			
Heart disease	43 (14.0%)	21 (20.0%)	0.140
Diabetes	33 (10.7%)	9 (8.6%)	0.530
Depression	40 (13.0%)	7 (6.7%)	0.078
CCQ total mean score	1.90 (±1.22)	2.39 (±1.30)	<0.0001
mMRC dyspnoea scale	2.78 (±1.32)	3.26 (±1.25)	0.001
			
*COPD stage*^a^			
I	62 (40.0%)	21 (25.6%)	Ref
II	67 (43.2%)	39 (47.6%)	0.094
III	24 (15.5%)	18 (22.0%)	0.048
IV	2 (1.3%)	4 (4.9%)	0.049

Patient characteristics at baseline in 2005, distributed over primary and secondary care. Data presented as numbers (percentages) or mean (s.d.).

Abbreviations: BMI, body mass index; CCQ, Clinical COPD Questionnaire; mMRC, modified Medical Research Council.

a Data available in a subgroup of 237 patients.

**Table 2:** Mean CCQ scores

*CCQ*	*2005*	*2012*	P*-value*
*Total mean score*			
All	2.03 (±1.26)	2.16 (±1.37)	0.011
Male	2.11 (±1.27)	2.12 (±1.29)	0.881
Female	1.97 (±1.25)	2.20 (±1.43)	0.003
			
*Symptoms*			
All	2.22 (±1.25)	2.32 (±1.35)	0.067
Male	2.45 (±1.29)	2.43 (±1.31)	0.870
Female	2.05 (±1.20)	2.24 (±1.38)	0.017
			
*Mental state*			
All	2.15 (±1.84)	2.05 (±1.77)	0.201
Male	2.00 (±1.84)	1.81 (±1.65)	0.076
Female	2.26 (±1.83)	2.23 (±1.83)	0.781
			
*Functional state*			
All	1.77 (±1.41)	2.06 (±1.61)	<0.0001
Male	1.83 (±1.35)	1.96 (±1.53)	0.155
Female	1.74 (±1.45)	2.13 (±1.67)	<0.0001

Total and domain CCQ scores in 2005 and 2012. Data presented as means (±s.d.).

Abbreviation: CCQ, Clinical COPD Questionnaire.

**Table 3:** Linear regression total CCQ mean score difference

*Independent variables 2005*	*CCQ difference between 2005 and 2012*					
	*Bivariable regression coefficient (95% CI)*	P*-value*	*Multivariable regression coefficient (95% CI) with adjustment for baseline CCQ*	P*-value*	*Multivariable regression coefficient (95% CI) with adjustment for baseline CCQ and confounders*	P*-value*
*Sex*						
Male	Ref		Ref		Ref	
Female	0.22 (0.002 to 0.43)	0.048	0.18 (−0.03 to 0.38)	0.088	0.13 (−0.09 to 0.33)	0.257
						
*Age*						
⩽60	−0.33 (−0.65 to −0.01)	0.046	−0.35 (−0.66 to 0.05)	0.024	−0.32 (−0.64 to −0.13)	0.042
61−70	−0.10 (−0.41 to 0.20)	0.507	−0.17 (−0.46 to 0.12)	0.247	−0.15 (−0.44 to 0.13)	0.290
>70	Ref		Ref		Ref	
						
*Educational level*						
Low	Ref		Ref		Ref	
High	−0.10 (−0.32 to 0.12)	0.381	−0.21 (−0.43 to −0.003)	0.047	−0.16 (−0.37 to 0.06)	0.151
						
*BMI*						
Underweight	0.35 (−0.06 to 0.77)	0.096	0.42 (0.02 to 0.81)	0.038	0.42 (0.03 to 0.81)	0.035
Normal weight	0.27 (0.04 to 0.50)	0.023	0.25 (0.03 to 0.47)	0.027	0.31 (0.09 to 0.53)	0.006
Overweight/obesity	Ref		Ref		Ref	
mMRC dyspnoea scale	−0.03 (−0.11 to 0.06)	0.538	0.26 (0.16 to 0.36)	<0.0001	0.26 (0.16 to 0.36)	<0.0001
						
*Subgroup*						
COPD stage^a^	0.05 (−0.32 to 0.43)	0.777	0.20 (0.02 to 0.38)	0.030	0.14 (−0.05 to 0.33)	0.161
						
*Excluded variables*						
Exacerbations in the previous 6 months						
0	Ref		Ref			
1	−0.08 (−0.39 to 0.23)	0.627	0.24 (−0.07 to 0.54)	0.125	—	
⩾2	−0.15 (−0.45 to 0.14)	0.310	0.26 (−0.04 to 0.56)	0.084	—	
Smoking						
Never	−0.20 (−0.64 to 0.23)	0.362	−0.20 (−0.61 to 0.22)	0.352	—	
Ex	−0.03 (−0.29 to 0.22)	0.792	−0.03 (−0.27 to 0.21)	0.817	—	
Occasional	0.23 (−0.27 to 0.73)	0.366	0.22 (−0.25 to 0.69)	0.366	—	
Current	Ref					
Heart disease	−0.13 (−0.43 to 0.16)	0.370	0.00 (−0.28 t0 0.28)	0.995	—	
Diabetes	0.09 (−0.26 to 0.44)	0.615	0.08 (−0.25 to 0.41)	0.638	—	
Depression	−0.27 (−0.60 to 0.07)	0.114	−0.13 (−0.45 to 0.19)	0.412	—	

Results from linear regression analyses with change in CCQ between 2005 and 2012 as dependent variable. The data are presented as regression coefficients (±95% confidence intervals). Multivariable analyses included both a conditional change model with adjustment for CCQ at baseline; and then adjustment for sex, age, all independent variables with a statistically significant association in the conditional change model; and CCQ at baseline.

Abbreviations: BMI, body mass index; CCQ, Clinical COPD Questionnaire; CI, confidence interval; mMRC, modified Medical Research Council.

a Analysis performed in a subgroup with *n*=237.

## Notes

BS is an Associate Editor of npj Primary Care Respiratory Medicine, but was not involved in the editorial review of, nor the decision to publish, this article. The other authors declare no conflict of interest.

## References

[bib1] From the Global Strategy for the Diagnosis, Management and Prevention of COPD, Global Initiative for Chronic Obstructive Lung Disease (GOLD) 2016. Available from: http://goldcopd.org/. 10.3760/cma.j.issn.0376-2491.2016.34.001 27667101

[bib2] Gudmundsson, G. et al. Risk factors for rehospitalisation in COPD: role of health status, anxiety and depression. Eur. Respir. J. 26: 414–419 (2005). 16135721 10.1183/09031936.05.00078504

[bib3] Gudmundsson, G. et al. Mortality in COPD patients discharged from hospital: the role of treatment and co-morbidity. Respir. Res. 7, 109 (2006). 16914029 10.1186/1465-9921-7-109 PMC1560381

[bib4] Sundh, J., Janson, C., Lisspers, K., Montgomery, S. & Stallberg, B. Clinical COPD Questionnaire score (CCQ) and mortality. Int. J. Chron. Obstruct. Pulmon. Dis. 7, 833–842 (2012). 23277739 10.2147/COPD.S38119 PMC3532021

[bib5] Wilke, S. et al. One-year change in health status and subsequent outcomes in COPD. Thorax 70: 420–425 (2015). 25782757 10.1136/thoraxjnl-2014-205697

[bib6] Vestbo, J. et al. Global Strategy for the Diagnosis, Management and Prevention of Chronic Obstructive Pulmonary Disease, GOLD Executive Summary. Am. J. Respir. Crit. Care Med. 187, 347–365 (2012). 22878278 10.1164/rccm.201204-0596PP

[bib7] Jones, P. W. et al. Development and first validation of the COPD Assessment Test. Eur. Respir. J. 34: 648–654 (2009). 19720809 10.1183/09031936.00102509

[bib8] van der Molen, T. et al. Development, validity and responsiveness of the Clinical COPD Questionnaire. Health Qual. Life Outcomes 1, 13 (2003). 12773199 10.1186/1477-7525-1-13 PMC156640

[bib9] van Dam van Isselt, E. F., Spruit, M., Groenewegen-Sipkema, K. H., Chavannes, N. H. & Achterberg, W. P. Health status measured by the Clinical COPD Questionnaire (CCQ) improves following post-acute pulmonary rehabilitation in patients with advanced COPD: a prospective observational study. NPJ Prim. Care Respir. Med. 24, 14007 (2014). 24842278 10.1038/npjpcrm.2014.7 PMC4373298

[bib10] Kon, S. S. et al. The Clinical COPD Questionnaire: response to pulmonary rehabilitation and minimal clinically important difference. Thorax 69: 793–798 (2014). 24149828 10.1136/thoraxjnl-2013-204119

[bib11] Fu, J. J., Gibson, P. G., Simpson, J. L. & McDonald, V. M. Longitudinal changes in clinical outcomes in older patients with asthma, COPD and asthma-COPD overlap syndrome. Respiration 87: 63–74 (2014). 24029561 10.1159/000352053

[bib12] Spencer, S., Calverley, P. M., Sherwood Burge, P. & Jones, P. W. Health status deterioration in patients with chronic obstructive pulmonary disease. Am. J. Respir. Crit. Care Med. 163: 122–128 (2001). 11208636 10.1164/ajrccm.163.1.2005009

[bib13] Oga, T. et al. Longitudinal deteriorations in patient reported outcomes in patients with COPD. Respir. Med. 101: 146–153 (2007). 16713225 10.1016/j.rmed.2006.04.001

[bib14] Peters, M. et al. Change in health status in long-term conditions over a one year period: a cohort survey using patient-reported outcome measures. Health Qual. Life Outcomes 12, 123 (2014). 25113415 10.1186/s12955-014-0123-2 PMC4243951

[bib15] Monteagudo, M. et al. Factors associated with changes in quality of life of COPD patients: a prospective study in primary care. Respir. Med. 107: 1589–1597 (2013). 23786889 10.1016/j.rmed.2013.05.009

[bib16] Jones, R. C. et al. Derivation and validation of a composite index of severity in chronic obstructive pulmonary disease: the DOSE Index. Am. J. Respir. Crit. Care Med. 180: 1189–1195 (2009). 19797160 10.1164/rccm.200902-0271OC

[bib17] Rolink M. et al. Using the DOSE index to predict changes in health status of patients with COPD: a prospective cohort study. Prim. Care Respir. J. 22: 169–174 (2013). 23538702 10.4104/pcrj.2013.00033 PMC6442782

[bib18] Celli, B. R. et al. The body-mass index, airflow obstruction, dyspnea, and exercise capacity index in chronic obstructive pulmonary disease. N. Engl. J. Med. 350: 1005–1012 (2004). 14999112 10.1056/NEJMoa021322

[bib19] Ferrari, R., Tanni, S. E., Caram, L. M., Naves, C. R. & Godoy, I. Predictors of health status do not change over three-year periods and exacerbation makes difference in chronic obstructive pulmonary disease. Health Qual. Life Outcomes 9, 112 (2011). 22152155 10.1186/1477-7525-9-112 PMC3254128

[bib20] Sood, A., Petersen, H., Meek, P. & Tesfaigzi, Y. Spirometry and health status worsen with weight gain in obese smokers but improve in normal-weight smokers. Am. J. Respir. Crit. Care Med. 189: 274–281 (2014). 24274793 10.1164/rccm.201306-1060OC PMC3977728

[bib21] Arne, M. et al. How often is diagnosis of COPD confirmed with spirometry? Respir. Med. 104: 550–556 (2010). 19931443 10.1016/j.rmed.2009.10.023

[bib22] Jones, P. W. et al. Health status in the TORCH study of COPD: treatment efficacy and other determinants of change. Respir. Res. 12, 71 (2011). 21627828 10.1186/1465-9921-12-71 PMC3117702

[bib23] Seemungal, T. A. et al. Effect of exacerbation on quality of life in patients with chronic obstructive pulmonary disease. Am. J. Respir. Crit. Care Med. 157: 1418–1422 (1998). 9603117 10.1164/ajrccm.157.5.9709032

[bib24] Stahl, E. et al. Health-related quality of life is related to COPD disease severity. Health Qual. Life Outcomes 3, 56 (2005). 16153294 10.1186/1477-7525-3-56 PMC1215504

[bib25] Sundh J. et al. Comparison of the COPD Assessment Test (CAT) and the Clinical COPD Questionnaire (CCQ) in a Clinical Population. COPD 13: 57–65 (2016). 26367315 10.3109/15412555.2015.1043426

[bib26] Ferreira, I. M., Brooks, D., White, J. & Goldstein, R. Nutritional supplementation for stable chronic obstructive pulmonary disease. Cochrane Database Syst. Rev. 12, CD000998 (2012). 23235577 10.1002/14651858.CD000998.pub3 PMC11742366

[bib27] Karner, C., Chong, J. & Poole, P. Tiotropium versus placebo for chronic obstructive pulmonary disease. Cochrane Database Syst. Rev. 7, CD009285 (2014). 25046211 10.1002/14651858.CD009285.pub3 PMC8934583

[bib28] Geake, J. B., Dabscheck, E. J., Wood-Baker, R. & Cates, C. J. Indacaterol, a once-daily beta2-agonist, versus twice-daily beta(2)-agonists or placebo for chronic obstructive pulmonary disease. Cochrane Database Syst. Rev. 1, CD010139 (2015). 25575340 10.1002/14651858.CD010139.pub2 PMC6464646

[bib29] McCarthy, B. et al. Pulmonary rehabilitation for chronic obstructive pulmonary disease. Cochrane Database Syst. Rev. 2, CD003793 (2015). 25705944 10.1002/14651858.CD003793.pub3 PMC10008021

[bib30] Sundh, J., Stallberg, B., Lisspers, K., Montgomery, S. M. & Janson, C. Co-morbidity, body mass index and quality of life in COPD using the Clinical COPD Questionnaire. COPD 8: 173–181 (2011). 21513436 10.3109/15412555.2011.560130

[bib31] Sundh, J., Janson, C., Lisspers, K., Stallberg, B. & Montgomery, S. The dyspnoea, obstruction, smoking, exacerbation (DOSE) index is predictive of mortality in COPD. Prim. Care Respir. J. 21: 295–301 (2012). 22786813 10.4104/pcrj.2012.00054 PMC6547953

[bib32] Sundh, J. et al. Management of COPD exacerbations in primary care: a clinical cohort study. Prim. Care Respir. J. 22: 393–399 (2013). 24114334 10.4104/pcrj.2013.00087 PMC6442855

[bib33] Sundh, J. et al. Comorbidity and health-related quality of life in patients with severe chronic obstructive pulmonary disease attending Swedish secondary care units. Int. J. Chron. Obstruct. Pulmon. Dis. 10, 173–183 (2015). 25653516 10.2147/COPD.S74645 PMC4310343

[bib34] Mahler, D. A. & Wells, C. K. Evaluation of clinical methods for rating dyspnea. Chest 93: 580–586 (1988). 3342669 10.1378/chest.93.3.580

[bib35] Quanjer, P. H. et al. Lung volumes and forced ventilatory flows. Report Working Party Standardization of Lung Function Tests, European Community for Steel and Coal. Official Statement of the European Respiratory Society. Eur. Respir. J. Suppl. 16, 5–40 (1993). 8499054

[bib36] Kocks, J. W. et al. Health status measurement in COPD: the minimal clinically important difference of the clinical COPD questionnaire. Respir. Res. 7, 62 (2006). 16603063 10.1186/1465-9921-7-62 PMC1508149

[bib37] Kon, S. S. et al. The Clinical COPD Questionnaire: response to pulmonary rehabilitation and minimal clinically important difference. Thorax 69, 793–798 (2013). 24149828 10.1136/thoraxjnl-2013-204119

